# Clinicopathological characteristics and imaging findings to identify adenomyosis‐related symptoms

**DOI:** 10.1002/rmb2.12409

**Published:** 2021-08-22

**Authors:** Shogo Imanaka, Hiroshi Shigetomi, Naoki Kawahara, Hiroshi Kobayashi

**Affiliations:** ^1^ Department of Obstetrics and Gynecology Nara Medical University Kashihara Japan; ^2^ Ms.Clinic MayOne Kashihara Japan; ^3^ Aska Ladies Clinic Nara Japan

**Keywords:** adenomyosis, dysmenorrhea, infertility, magnetic resonance imaging, menorrhagia

## Abstract

**Purpose:**

The study aims to identify the clinicopathological risk factors and magnetic resonance (MR) imaging findings for adenomyosis‐related symptoms, including menorrhagia, dysmenorrhea, and infertility.

**Methods:**

This was an observation‐based cross‐sectional study using data from the adenomyosis cohort study. The authors evaluated the clinicopathological variables and various MR imaging findings.

**Results:**

Two hundred twenty patients with histologically confirmed adenomyosis were included in this study. Multivariate analysis showed that a middle/retroflexed uterus and adenomyosis lesions of 21 mm or more were significant independent predictors of dysmenorrhea. The history of dysmenorrhea and the maximum length from the cervix to the uterine fundus ≥103 mm were independent risk factors of menorrhagia. One of the key factors associated with non‐infertility included the absence of deep infiltrating endometriosis (DIE) and/or superficial peritoneal disease (SUP).

**Conclusions:**

This study identified clinicopathological risk factors and imaging findings associated with adenomyosis‐related symptoms. The maximum length from the cervix to the uterine fundus and adenomyosis lesion thickness are independent predictors for the presence of menorrhagia and dysmenorrhea, respectively. Infertility may be associated with the coexistence of endometriosis rather than adenomyosis itself. This result is from an analysis of a small number of infertility patients and requires further study.

## 1 INTRODUCTION

Adenomyosis is a common benign gynecological disorder where endometrial tissue invades the uterine myometrium, causing myometrial hypertrophy.^1^, ^2^ It has been diagnosed by surgical procedure, such as hysterectomy.^3^ Adenomyosis may affect 20% of the female population and is frequently observed in premenopausal and perimenopausal women.^1^ However, this disorder can also be diagnosed in young women who are symptomatic using noninvasive modalities such as transvaginal ultrasound and/or magnetic resonance (MR) imaging.^3^, ^4^, ^5^ This condition is associated with a wide variety of symptoms presenting as menorrhagia, dysmenorrhea, and chronic pelvic pain and infertility,^5^, ^6^, ^7^ but some women are asymptomatic.^2^, ^3^ Many researchers are interested in why patients have different symptoms. Menorrhagia and metrorrhagia are the main clinical manifestations.^8^ In many cases, both symptoms occur simultaneously.^7^ In clinical practice, physicians experience that diffuse adenomyosis causes more severe menstrual symptoms compared with focal localized adenomyosis.^9^ In addition, the disease has a potential negative impact on female fertility.^8^ Accumulating evidence has shown that adenomyosis is associated with the risk of pregnancy outcome and obstetric complications.^10^, ^11^ Many patients with adenomyosis also have endometriosis, so it is difficult to determine whether adenomyosis is the only cause of infertility.^12^ At this time, no clinicopathological characteristics and imaging findings associated with infertility have been identified. Adenomyosis is a heterogeneous group of conditions that include a range of clinical presentations, and its biological behavior remains incompletely understood.^6^ In particular, the prediction of the onset of infertility is challenging due to their heterogeneity and various confounding factors.

Therefore, the study aims to identify the clinicopathological risk factors and imaging findings for adenomyosis‐related symptoms, including menorrhagia, dysmenorrhea, and infertility in patients who were histologically diagnosed with adenomyosis in a single university hospital.

## 2 METHODS

### 2.1 Patient selection and data collection

The study was approved by the medical ethics committee of the Nara Medical University (reference nos. 541, 951, and 2295). Written informed consent was obtained from each patient.

A single‐center prospective cohort (DoG‐NaMe) study was conducted by collecting data from patients admitted to the Department of Gynecology, Nara Medical University Hospital, Kashihara, Japan, from January 2008 to December 2020. The DoG‐NaMe study consists of an endometriosis cohort, an adenomyosis cohort, and an ovarian cancer cohort. Women scheduled for surgery were primarily enrolled. We performed an observational cross‐sectional study using data from the adenomyosis cohort study. Participants underwent surgery or active surveillance (including some hormone therapy) to manage adenomyosis. Indications for surgical treatment are progressive anemia (8.0 g/dl or less of hemoglobin), exacerbation of clinical symptoms that cause abdominal compression and discomfort in daily life, severe pelvic pain that is difficult to control, and others. The following inclusion criteria were used (1) patients undergoing surgery with removal of lesions for histological evaluation; (2) patients with pathological confirmation of adenomyosis; and (3) patients who underwent magnetic resonance imaging (MRI) examinations prior to surgery. The criteria for exclusion were as follows: (1) age below 20 years; (2) active surveillance only; (3) hormone therapy only; (4) women during menstruation; (5) postmenopausal women; (6) women coexisting with malignancies; and (7) incomplete data. Women with a history of hormone therapy were not excluded. All participants were recommended to undergo MRI after routine transvaginal ultrasonography (TVS) for preoperative evaluation of adenomyosis. MRI scanning was performed except during the menstrual phase. TVS was performed by experienced operators with a special interest in gynecological diseases with a single ultrasound system (Voluson E8; GE Healthcare, Tokyo, Japan) using a transvaginal transducer (5–7.5 MHz). MRI was obtained on a 3T system using T1W and T2W sequences (Magnetom Verio, Siemens Healthcare, Erlangen, Germany). The protocol of our MRI examination was performed as described previously.^13^ Imaging diagnosis, including TVS and MRI, was completed within 4 weeks prior to surgery.

Demographic, clinicopathological, and imaging data were retrieved from the electronic medical records linked to the centralized computer system. Patient's age at surgery, gravidity, parity, a history of cesarean sections and induced abortion, body mass index (BMI), adenomyosis‐related symptoms, including dysmenorrhea/menstrual pain, menorrhagia and infertility, preoperative hemoglobin levels, and preoperative serum CA125 levels were collected. The following lesion‐related parameters were measured based on MRI: uterine size (maximum length from cervix to uterine fundus [cavity longitudinal distance]), myometrial thickness (the thickest myometrial layer [either] and the thickest myometrial layer [sum]), adenomyotic lesion thickness, and proportion of anteflexed and midline/retroflexed uterus. A detailed description of these indicators can be found in reference 14. In addition, adenomyosis was classified as follows according to ref.^15^, ^16^ based on the affected area and the degree of myometrial infiltration. The intrinsic type is defined as adenomyosis that occurs in the uterine inner layer without affecting the outer structures of the myometrium. The extrinsic type is defined as adenomyosis that occurs in the uterine outer layer without affecting the inner structures. If either of the two gynecologists diagnosed the patient as neither intrinsic adenomyosis nor extrinsic adenomyosis, she was classified as "unclassifiable or unidentifiable."

### 2.2 Definition of adenomyosis‐related symptoms: dysmenorrhea, menorrhagia, and infertility

A detailed definition of dysmenorrhea and menorrhagia can also be found in Reference 14. All patients were divided into two groups in terms of the presence and severity of dysmenorrhea and menorrhagia. In this study, “moderate pain” and “severe pain” were classified as having dysmenorrhea using the Numeric Rating Scale (NRS‐11).^17^ Menorrhagia was categorized according to the Mayo Clinic definition.^18^

There were 2 types of patients in the infertility group: Patients who failed to achieve a clinical pregnancy following ≥12 months of regular unprotected sexual intercourse^19^ and those who have already been treated at fertility hospitals. When a woman became pregnant with common fertility treatments such as timed intercourse, she was classified as “not infertile”. To better clarify the risk factors, the definition of infertility in this study was limited to patients who underwent in vitro fertilization. Male factor infertility was excluded. Women who were unmarried, divorced, or did not wish to become pregnant were excluded from this analysis.

### 2.3 Quantification of serum CA125

Blood samples were obtained from all participants to determine serum/plasma levels of hemoglobin and CA125 within 4 weeks prior to surgery. Blood sampling was performed except during the menstrual phase. Samples were centrifuged at 1500×*g* for 10 min at 4℃, separated into serum and plasma, and stored at −20℃. Serum CA125 concentrations were determined using an electrochemiluminescence Elecsys immunoassay (ECLIA) (Roche Diagnostics, Salzburg, Austria) at Nara Medical University Hospital.

### 2.4 Statistical analysis

SPSS 25.0 (SPSS Inc., Chicago, IL) statistical software was used for the statistical analysis. The data are presented as mean ± SD or median (range) for the continuous and the categorical variables, respectively. The normality of the data was examined using the Shapiro‐Wilk test, and based on the results, differences in parameters between the two groups were analyzed using a parametric or nonparametric test. Continuous variables were compared with Student's *t* test or Mann‐Whitney U test if the variables were not normally distributed. Categorical variables were compared using chi‐square test. Receiver operating characteristic (ROC) analysis and the area under curve (AUC) were used to identify the sensitivity and specificity of each parameter cutoff point. The Youden index was used to determine optimal cutoff values. A multivariable regression model was employed after testing for multicollinearity. The multicollinearity relationships between pairs of variables were examined using a correlation test. The existence of multicollinearity was determined by high values of correlation coefficient (>0.7). Univariate and multivariate logistic regression models were used to evaluate these relationships between the parameters and the risk factors of adenomyosis‐related symptoms; then, odds ratio (ORs) and 95% confidence intervals (CIs) were calculated. Differences with *p* <  0.05 were considered statistically significant.

## 3 RESULTS

**FIGURE 1:**
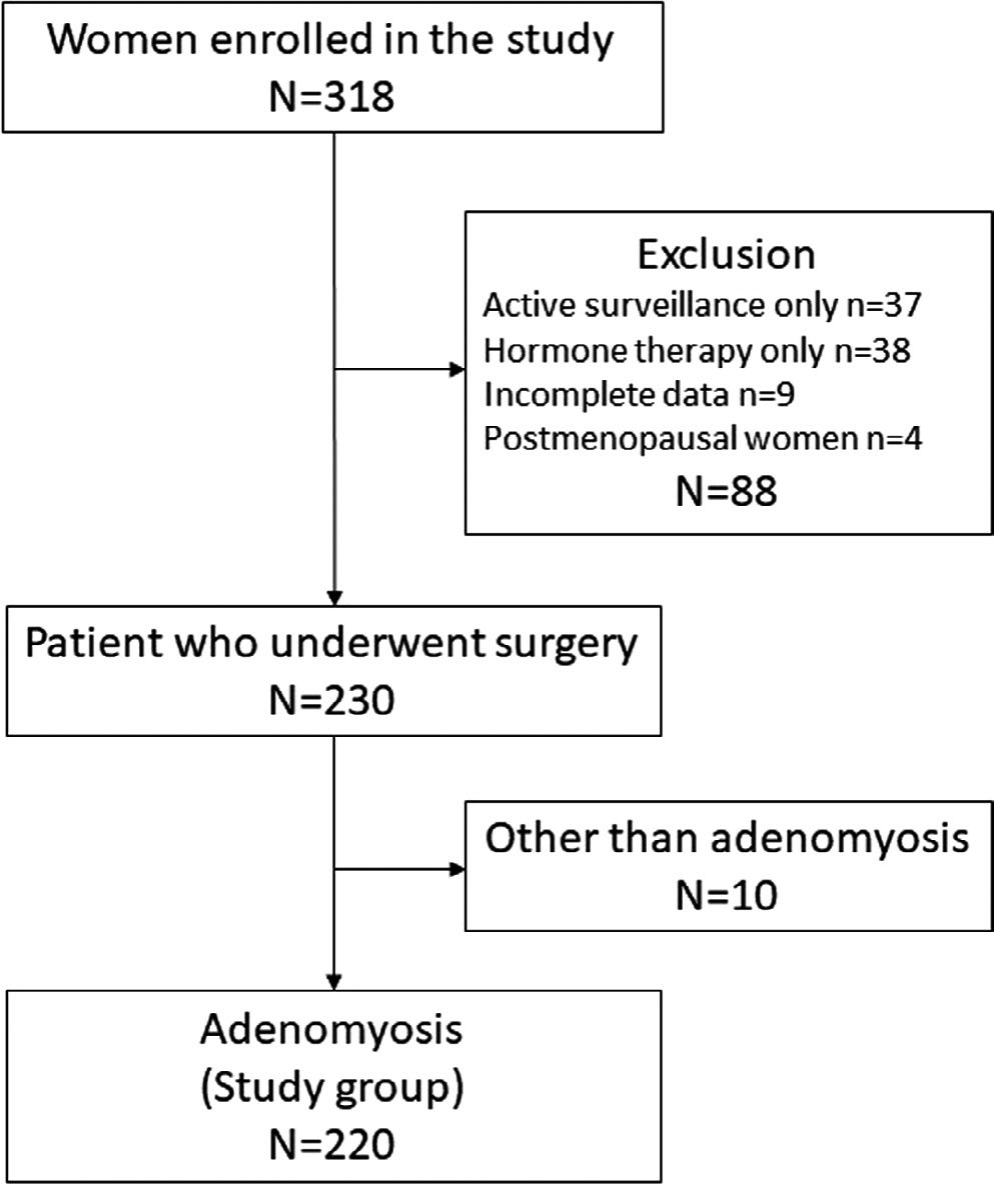
Flow diagram of patient selection and exclusion

Three hundred eighteen women participated in the adenomyosis cohort study. After 98 women were excluded because of exclusion criteria (*n* = 88) and no pathological confirmation of adenomyosis (*n* = 10), the 220 patients were analyzed in this study. The clinicopathological characteristics and treatment strategies of the 220 patients reported here are detailed in a recently published paper.^14^ The outline of the treatment is as follows. Based on the severity of the present disease, 140 women underwent surgical resection as the first‐line treatment and 130 women were histologically confirmed to have adenomyosis. Of the 128 women who received the pharmacologic management as the first‐line option, 90 women eventually underwent surgical resection, mainly due to the insufficient effectiveness of current hormone therapies. The reasons for switching from hormone therapy to surgery were as follows: (1) progressive anemia due to recurrence of persistent abnormal uterine bleeding (*n* = 64), (2) exacerbation of abdominal compression symptoms (*n* = 11), (3) severe pelvic pain (*n* = 5), and (4) side effects of drugs including mental health problems (*n* = 10). Seventy‐three women (33.2%) exhibited intrinsic adenomyosis, 77 women (35.0%) exhibited extrinsic adenomyosis, and 70 women (31.8%) exhibited unclassifiable phenotype based on the MRI. Figure 1 illustrates the detailed selection process for study design.

**FIGURE 2:**
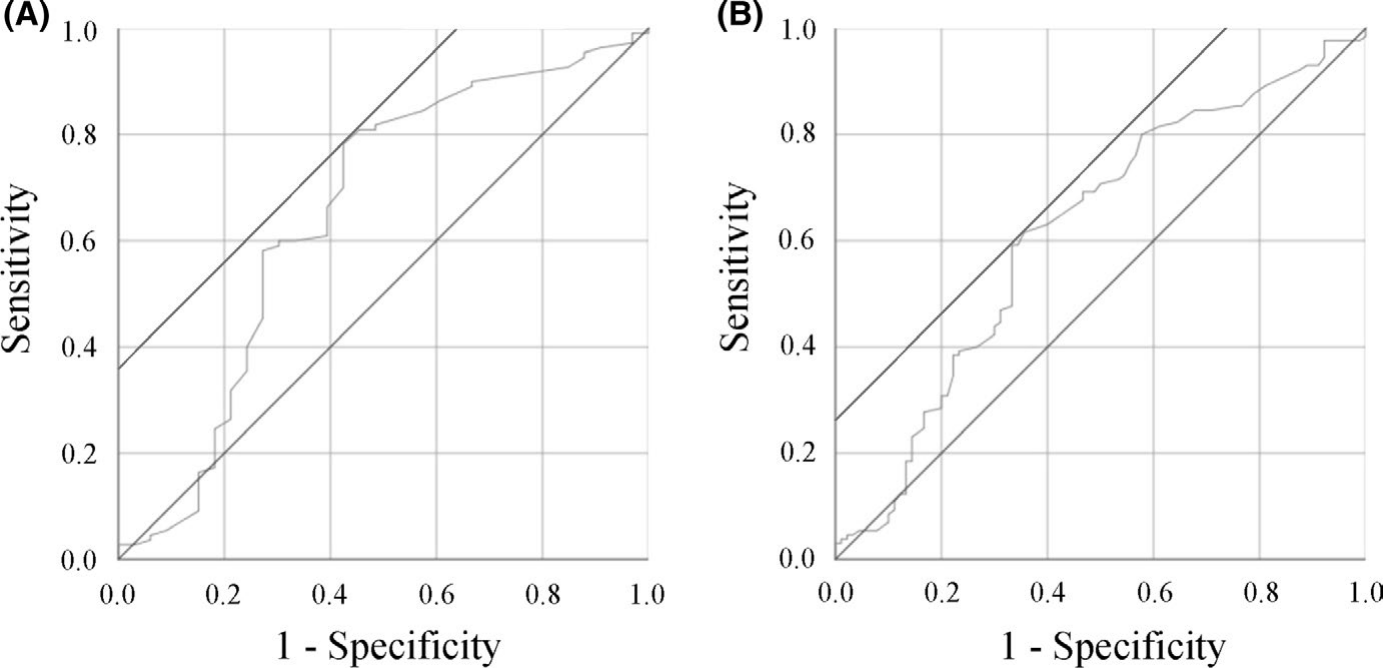
Receiver operating characteristic (ROC) analysis for indicators that affect dysmenorrhea (A) and menorrhagia (B). (A) ROC curve analysis to evaluate the cutoff value of adenomyosis lesion thickness for dysmenorrhea. (B) ROC curve analysis to evaluate the cutoff value of maximum length from cervix to uterine fundus for menorrhagia

Of the 220 women, 68 and 152 were painless + mild (described as “Absence”) and moderate + severe (described as “Presence”) for the severity of dysmenorrhea, respectively. The prevalence of patients with dysmenorrhea was 69.1% (*n* = 152). To identify the potential risk factors of the presence of dysmenorrhea, Youden index was measured to determine the optimal cutoff threshold. ROC curves were created to identify the best cutoff value, sensitivity and specificity, area under the curve (AUC), and p‐value of each variable that affects dysmenorrhea (Table 1). The optimal cutoff values computed using the ROC were 43 years for age, 30 mm for the thickest myometrium [either], 57 mm for the thickest myometrium [sum], 21 mm for the thickest adenomyosis lesion, and 31 U/ml for serum CA125 level. For example, the optimal cutoff value for adenomyotic lesion thickness in distinguishing between women with dysmenorrhea and women without dysmenorrhea was 21 mm with a sensitivity of 78.2% and specificity of 57.6% with an AUC of 0.652 (95% CI 0.533–0.772, *p* = 0.008). Figure 2A shows ROC analysis for an indicator (adenomyosis lesion thickness) that affects dysmenorrhea. The possible risk factors for predicting the presence of dysmenorrhea were categorized into 2 groups for comparison in the univariate analysis, and the significant factors were further evaluated in the multivariate analysis as noted in Table 2. Multicollinearity between the variables often results in model overfitting in a multivariate regression test, which in turn leads to imprecise estimation. Multicollinearity was identified between the variables, including gravidity and parity, coexistence of OMA and coexistence of DIE and/or SUP, the thickest myometrium [either] and the thickest myometrium [sum], and the thickest myometrium [either] and the thickest adenomyosis lesion. In this study as well, multicollinearity affected the estimates from the multivariate regression models. Therefore, the three variables (gravidity, coexistence of OMA, and the thickest myometrium [either]) were removed in a multivariate regression model. If only one of the two variables with multicollinearity was selected by univariate analysis, this variable was chosen for multivariate analysis. On univariate analysis, midline/retroflexed uterus (*p*=0.019), the thickest adenomyosis lesion ≥21 mm (*p* < 0.001), the thickest myometrium [either] ≥30 mm (*p* < 0.001), the thickest myometrium [sum] ≥57 mm (*p* < 0.001), menorrhagia (*p* < 0.001), age at surgery ≥43 years (*p* = 0.001), CA125 ≥30.5 U/ml (*p* = 0.005), and coexistence of OMA (*p* = 0.032) were significantly more common in women with dysmenorrhea than in those without (Table 2). The median age of patients at surgery was 3 years younger in the dysmenorrhea group than in the painless group (42.9 ± 5.5 years vs. 45.3 ± 5.6 years; *p* = 0.003). We investigated the impact of the adenomyosis phenotype on dysmenorrhea, but there was no significant difference in the proportion of adenomyosis phenotype between the two groups (*p* = 0.371). Multivariate analysis revealed that only the midline/retroflexed uterus and the thickest adenomyosis lesion ≥21 mm were independent risk factors that affect dysmenorrhea with the ORs of 7.494 (*p* = 0.026) and 4.332 (*p* = 0.047), respectively (Table 2).

**TABLE 1:** Optimal cutoff values for each variable determined by ROC analysis based on the presence of dysmenorrhea in patients with adenomyosis

Factor	Cutoff	Sensitivity	1‐Specificity	AUC	50% CI	*p*‐Value
Age	42.5	0.794	0.559	0.632	0.551–0.713	0.002
The thickest myometrial layer [either]	29.5	0.693	0.353	0.681	0.601–0.762	<0.001
The thickest myometrial layer [sum]	56.5	0.563	0.265	0.619	0.536–0.702	0.005
Adenomyotic lesion thickness	20.5	0.782	0.424	0.652	0.533–0.772	0.008
CA125	30.5	0.641	0.364	0.625	0.519–0.732	0.027

**TABLE 2:** Univariate and multivariate analyses to identify risk factors affecting dysmenorrhea

Factor	Category	No. of patients							
		Dysmenorrhea		Univariate analysis			Multivariate analysis		
		Absence	Presence	OR	95% CI	P‐value	OR	95% CI	P‐value
Anteflexed and midline/retroflexed	Absence	51	91	2.204	1.137–4.275	0.019	7.494	1.271–44.184	0.026
	Presence	15	59						
The thickest adenomyosis lesion	<20.5	19	24	4.863	2.130–11.102	<0.001	4.332	1.021–18.381	0.047
	≥20.5	14	86						
The thickest myometrium [either]	<29.5	44	46	4.145	2.260–7.602	<0.001			
	≥29.5	24	104						
The thickest myometrium [sum]	<56.5	50	66	3.577	1.910–6.700	<0.001			
	≥56.5	18	85						
Menorrhagia	Absence	49	41	6.982	3.684–13.234	<0.001			
	Presence	19	111						
Age at surgery	≥43	54	85	3.040	1.557–5.938	0.001			
	<43	14	67						
CA125	<30.5	28	23	3.120	1.403–6.934	0.005			
	≥30.5	16	41						
Coexistence of OMA	Absence	53	96	2.061	1.064–3.993	0.032			
	Presence	15	56						

Next, of the 220 patients, 130 (59.1%) women had menorrhagia. As shown in Table 3, the optimal cutoff values were 103 mm for the maximum length from the cervix to the uterine fundus, 29 mm for the thickest myometrium [either], and 61 mm for the thickest myometrium [sum]. Figure 2B shows ROC analysis for an indicator (maximum length from cervix to uterine fundus) that affects menorrhagia. The ROC curve was drawn based on the maximum length from cervix to uterine fundus and the presence of menorrhagia, from which the optimal cutoff values are 103 mm, sensitivity 59.2%, specificity 66.7%, and AUC 0.618 (95% CI, 0.541–0.695, *p* = 0.003). The univariate analysis indicated that the presence of dysmenorrhea (*p* < 0.001), maximum length from cervix to uterine fundus (*p* < 0.001), the thickest myometrium [either] (*p* = 0.001), and the thickest myometrium [sum] (*p* = 0.002) were significantly associated with a history of menorrhagia as described in Table 4. There were no significant differences in terms of other variables between the two groups. Multivariate analysis indicated that the presence of dysmenorrhea and maximum length from cervix to uterine fundus ≥103 mm were independent potential risk factors for the presence of menorrhagia, with the respective ORs of 6.668 (*p* < 0.001) and 2.711 (*p* = 0.001) (Table 4).

**TABLE 3:** Optimal cutoff values for each variable determined by ROC analysis based on the presence of menorrhagia in patients with adenomyosis

Factor	Cutoff	Sensitivity	1‐Specificity	AUC	50% CI	*p*‐Value
Maximum length from cervix to uterine fundus, mm	102.5	0.592	0.333	0.618	0.541–0.695	0.003
The thickest myometrium [either]	28.5	0.705	0.472	0.598	0.520–0.676	0.014
The thickest myometrium [sum]	60.5	0.504	0.289	0.582	0.504–0.660	0.039

**TABLE 4:** Univariate and multivariate analyses to identify risk factors affecting menorrhagia

Factor	Category	No. of patients							
		Menorrhagia		Univariate analysis			Multivariate analysis		
		Absence	Presence	OR	95% CI	*p*‐Value	OR	95% CI	*p*‐Value
Dysmenorrhea	Absence	49	19	6.982	3.684–13.234	<0.001	6.668	3.468–12.896	<0.001
	Presence	41	111						
Maximum length from cervix to uterine fundus, mm	<102.5	60	53	2.906	1.658–5.091	<0.001	2.711	1.468–5.010	0.001
	≥102.5	30	77						
The thickest myometrium [either]	<28.5	48	39	2.667	1.525–4.663	0.001			
	≥28.5	42	91						
The thickest myometrium [sum]	<60.5	64	65	2.462	1.391–4.357	0.002			
	≥60.5	26	65						

Fifty women were excluded from fertility analysis because they were unmarried, divorced, or did not want to become pregnant. Of the 170 patients, 11 (4.9%) women underwent in vitro fertilization. The optimal cutoff values were 41 years for age, 1.5 for gravidity, and 1.5 for parity (Table 5). The absence of a history of infertility was significantly associated with older age (*p* < 0.001), less frequent OMA (*p* = 0.004) and DIE/SUP (*p* = 0.008), a higher gravidity (*p* = 0.015), and a higher parity (*p* = 0.021) by univariate analysis (Table 6). The multivariate analysis revealed that 1) women with adenomyosis over 41 years were associated with the absence of infertility (*p* = 0.003), and 2) the absence of DIE and/or SUP was associated with not being infertile (*p* = 0.026) (Table 6).

**TABLE 5:** Optimal cutoff values for each variable determined by ROC analysis based on the presence of infertility in patients with adenomyosis

Factor	Cutoff	Sensitivity	1‐Specificity	AUC	50% CI	*p*‐Value
Age at surgery	40.5	0.811	0.182	0.824	0.718–0.930	<0.001
Gravidity	1.5	0.673	0.273	0.723	0.569–0.877	0.013
Parity	1.5	0.535	0.091	0.751	0.634–0.868	0.005

**TABLE 6:** Univariate and multivariate analyses to identify risk factors affecting infertility.

Factor	Category	No. of patients							
		Infertility		Univariate analysis			Multivariate analysis		
		Absence	Presence	OR	95% CI	*p*‐Value	OR	95% CI	*p*‐Value
Age at surgery	≥41	129	2	19.350	3.974–94.211	<0.001	12.104	2.294–63.871	0.003
	<41	30	9						
Coexistence of DIE and/or SUP	Absence	108	2	9.000	1.797–45.087	0.008	6.741	1.262–35.992	0.026
	Presence	42	7						
Gravidity	≥2	107	3	5.487	1.398–21.543	0.015			
	≤1	52	8						
Parity	≥2	85	1	11.486	1.436–91.862	0.021			
	≤1	74	10						
Coexistence of OMA	Absence	118	3	7.675	1.943–30.313	0.004			
	Presence	41	8						

## 4 DISCUSSION

We attempted to identify the potential clinicopathological risk factors and imaging findings affecting the adenomyosis‐related symptoms, including pain, bleeding, and infertility. The selection of the study population was based on strict inclusion and exclusion criteria. In our cohort study, the mean age at surgery was 43.5 years, with moderate‐to‐severe dysmenorrhea and menorrhagia of 152 (69.1%) and 130 (59.1%), respectively. Among them, the coexistence of OMA, DIE, and SUP was 71 (32.3%), 58 (26.4%), and 53 (24.1%) cases, respectively, but there was considerable overlap. Only a few reports have simultaneously examined the detailed risk factors for dysmenorrhea, menorrhagia, and infertility associated with adenomyosis.^15^, ^20^

First, previous studies have shown that patients with adenomyosis are more likely to have dysmenorrhea.^21^, ^22^, ^23^, ^24^ Our study found that the risk of dysmenorrhea was markedly associated with the degree of menorrhagia and that patients with dysmenorrhea were younger, coexisted more with OMA, had higher CA125 levels, had a thicker myometrial layer, and had a more middle/retroflexed uterus compared with patients without dysmenorrhea (Table 2). Independent risk factors for dysmenorrhea were women with a middle/retroflexed uterus and those with adenomyosis lesions of 21 mm or more. The coexistence of endometriosis is associated with adenomyosis‐related dysmenorrhea, but this risk factor was lost in the multivariate model. Women with adenomyosis with a middle/retroflexed uterus were 7.5 times more at risk of developing dysmenorrhea than those with an anteflexed uterus (Table 2). This is the first report of a link between adenomyosis‐related dysmenorrhea and retroflexed uterus. Previously, the particular endometrial shape associated with adenomyosis was named the "question mark sign," which is a sign that suggests the presence of posterior DIE.^25^ Adenomyosis with a retroverted uterus may indicate possible adhesions between the uterus and posterior structures in close proximity. In addition, the presence of dysmenorrhea is more strongly associated with adenomyosis lesion thickness than with coexistence of endometriosis. This suggests that dysmenorrhea symptoms can be further exacerbated by diffuse adenomyosis, which is prone to widespread intralesional bleeding. When adenomyotic lesions penetrate deep into the myometrial layer, dysmenorrhea becomes more severe and outweighs the impact of endometriosis‐dependent pain. We provided a concrete indicator that dysmenorrhea occurs when the adenomyosis lesion is 21 mm or thicker. Our results were supported by some of the previously published papers. Severe dysmenorrhea was significantly associated with younger age.^7^ The greater the number of ultrasound imaging findings suggestive of adenomyosis, the more severe the symptoms.^26^, ^27^, ^28^ The rate of severe dysmenorrhea increased steadily depending on the depth of myometrial involvement (shallow and deep myometrial involvement; 4.3% vs. 83.3%).^3^, ^29^ Sammour et al. (2002) found a significant correlation between dysmenorrhea, but not menorrhagia, and the depth of adenomyosis.^30^ Women with a large number or high density of ectopic endometrial glands have been reported to suffer from more severe dysmenorrhea.^3^, ^29^, ^31^ However, Exacoustos et al. reported that there was no statistically significant difference between women with diffuse adenomyosis and those with focal disease regarding the presence and severity of dyspareunia and dysmenorrhea.^24^ Moreover, several researchers have hypothesized that patients with extrinsic adenomyosis are more likely to have endometriosis, so the degree of dysmenorrhea can be more severe than patients with intrinsic adenomyosis.^15^, ^32^ Bourdon et al. reported that although adenomyosis phenotypes such as external or internal adenomyosis have different clinical characteristics, no differences were noted in terms of pain symptoms.^20^ Due to the lack of an internationally universal definition of intrinsic and extrinsic adenomyosis, and diffuse and focal disease, we were unable to compare our results with other studies to assess whether dysmenorrhea is affected by the location and spread of adenomyosis foci.

Second, our results showed that the history of dysmenorrhea and the maximum length from the cervix to the uterine fundus are independent risk factors for predicting the severity of menorrhagia. We provided a concrete indicator that menorrhagia increases when the maximum length from the cervix to the uterine fundus is 103 mm or more. This suggests that compared with a barrel‐shaped uterus with a thick myometrium, women with tall adenomyosis can cause more severe menorrhagia. Our results are in agreement with the findings of previous studies. For example, the severity of menorrhagia did not correlate with uterine myometrial thickness.^3^, ^29^ The size of the uterus did not differ significantly between symptomatic and asymptomatic women^7^, ^33^ There was no difference in the number of foci in women with and without menorrhagia.^31^ On the other hand, Exacoustos et al. reported that women with ultrasound diagnosis of diffuse adenomyosis had heavier menstrual bleeding than women with focal disease^24^ Pistofidis et al. classified adenomyosis into four different phenotypes, diffuse, sclerotic, nodular, and cystic and examined the frequency of menorrhagia.^34^ They showed that patients with diffuse type (84%) had a significantly higher frequency of menorrhagia than those with sclerotic (44%) and nodular (37%) types (*p* = 0.025 and *p* = 0.001, respectively).^34^ Thus, the results of some researchers were different from ours. In addition, patients with shallow myometrial involvement were more likely to suffer from menorrhagia than those with deeper disease (60% vs. 42%, respectively).^3^, ^29^ Bourdon et al. recently published that heavy menstrual bleeding was more common in women with internal adenomyosis compared with those with external adenomyosis.^20^ Continuous dienogest therapy caused unpredictable bleeding in about one‐third of patients with endogenous adenomyosis.^35^ The authors considered that in patients with intrinsic adenomyosis, microvessels arising from adenomyotic lesions continue to the eutopic endometrium, leading to heavy bleeding.^35^ It is easy to imagine that the longer the uterus with intrinsic adenomyosis, the more severe the menorrhagia.^24^, ^34^ Our data could not conclude that patients with intrinsic adenomyosis had more bleeding than patients with extrinsic adenomyosis. In this study, the presence of dysmenorrhea had a profound effect on menorrhagia, with an odds ratio of 6.668 (Table 4), which may negate the effects of other predictors, including the adenomyosis phenotype.

Third, many researchers believed that patients with adenomyosis have significantly lower pregnancy and implantation rates,^36^, ^37^ and higher miscarriage rates,^38^ adversely affect IVF results, increase the risk of obstetric complications such as preterm birth and premature rupture of membrane, compared to women without adenomyosis.^4^, ^11^, ^38^ However, it is not clear which phenotype of adenomyosis causes infertility. Univariate analysis revealed that infertile women were younger, had lower gravity and parity, and more frequent coexistence of OMA and DIE/SUP compared with fertile women. It is not surprising that infertile patients are young, have low gravity, and have low parity. Multivariate analysis showed that age ≥41 years and the absence of DIE and/or SUP were independent predictors of non‐infertility (Table 6). Infertile women who underwent surgery (*n* = 11) were 6 years younger than non‐infertility women who underwent surgery (*n* = 159) (38.6 ± 4.5 vs. 44.9 ± 5.1, *p* < 0.001). Women with infertility may have undergone surgical treatment at a young age due to frequent visits to medical institutions. Due to the small number of infertility patients and the very high odds ratios of age, parity, and DIE, no significant association was found between the adenomyosis phenotype and infertility. Bourdon et al. noted that focal adenomyosis of the outer myometrium (FAOM) was more frequently found in women with a primary infertility than in non‐infertile women.^20^, ^39^ Women with FAOM are more likely to suffer from endometriosis,^20^, ^39^ which can lead to an increased incidence of infertility. On the other hand, some reports demonstrated that there was no statistical difference in pregnancy rates between patients with localized adenomyosis and diffuse adenomyosis.^36^, ^40^ There is still limited evidence as to whether infertility depends on the phenotype of adenomyosis or is secondary to the associated endometriosis. Furthermore, even considering multicollinearity, the absence of endometriosis (DIE and/or SUP, or OMA) was found to be an independent factor predicting non‐infertility. Infertility may be associated with the coexistence of endometriosis rather than adenomyosis itself.

Finally, the strength of our study is the analysis of data from a large number of patients with histologically confirmed adenomyosis in a single university hospital. Despite this strength, there are some weaknesses. Not only clinical information about the patient's age and comorbidities such as endometriosis but also detailed imaging diagnosis on the phenotype, location, and extent of the lesion are needed to predict the presence and severity of symptoms associated with adenomyosis. Adenomyosis phenotypes, such as "intrinsic or extrinsic" and "diffuse or focal," help to consider the origin and spread of the disease and the severity of the symptoms. However, our results cannot be compared to those for other due to the lack of internationally unified definitions of diffuse and focal adenomyosis, intrinsic and extrinsic adenomyosis, and internal and external adenomyosis.^16^ Multicenter, prospective clinical trials should be conducted to identify the risk factors of adenomyosis symptoms using internationally unified classifications. Another weakness of this study is the timing of MRI scans in infertile women. MRI scans should be performed when the patient wishes to become pregnant, not just before surgery. However, many patients were diagnosed with adenomyosis by transvaginal ultrasound at the beginning of fertility treatment and then underwent MRI after the condition has progressed. Therefore, it remains questionable whether preoperative MRI findings really reflect an association with infertility.

In conclusion, the clinicopathological risk factors and imaging findings for adenomyosis‐related symptoms were identified. The maximum length from the cervix to the uterine fundus (≥103 mm) and adenomyosis lesion thickness (≥21 mm) are associated with the presence of bleeding and pain, respectively. Adenomyosis‐related infertility may be associated with the coexistence of endometriosis. However, due to the small number of infertility patients and the limited value of clinical research, further research is required. The results of this study will help plan future multicenter studies that predict the severity of adenomyosis symptoms by quantifying anatomical structures based on MR imaging features.

## CONFLICT OF INTEREST

The authors declare no conflict of interest. Human and Animal Rights: The study was conducted under the guidelines that had been approved by the medical ethics committee of the Nara Medical University (reference nos. 541, 951, and 2295). This article does not contain any study with animal participants that have been performed by any of the authors.

## AUTHOR CONTRIBUTIONS

Hiroshi Shigetomi and Hiroshi Kobayashi contributed to the study conception and design. Shogo Imanaka and Hiroshi Shigetomi collected patient information from electronic medical records and compiled it into an Excel sheet. Shogo Imanaka and Hiroshi Shigetomi performed the literature search and collected data on adenomyosis using the Web database. The data analysis was performed by Shogo Imanaka and Naoki Kawahara. The first draft of the manuscript was written by Hiroshi Kobayashi. The final version of the manuscript has been read and approved by all authors.

## Data Availability

The datasets generated during the current study are available from Hiroshi Kobayashi on reasonable request. Written informed consent was obtained from each patient. Human rights statements and informed consent: All procedures followed were in accordance with the ethical standards of the responsible committee on human experimentation (institutional and national) and with the Helsinki Declaration of 1964 and its later amendments.
